# Hippo-YAP/TAZ signaling in breast cancer: Reciprocal regulation of microRNAs and implications in precision medicine

**DOI:** 10.1016/j.gendis.2023.01.017

**Published:** 2023-03-23

**Authors:** Farzad Sadri, Seyede fatemeh Hosseini, Zohreh Rezaei, Mohammad Fereidouni

**Affiliations:** a Student Research Committee, Birjand University of Medical Sciences, Birjand 9717853577, Iran; b Tabas School of Nursing, Birjand University of Medical Sciences, Birjand 9717853577, Iran; c Department of Biology, University of Sistan and Baluchestan, Zahedan 9816745785, Iran; d Cellular and Molecular Research Center, Birjand University of Medical Sciences, Birjand 9717853577, Iran

**Keywords:** Breast cancer, Hippo signaling, Large tumor suppressor kinase 1, microRNAs, PDZ-binding motif, Yes-associated protein

## Abstract

Breast cancer is a molecularly heterogeneous disease and the most common female malignancy. In recent years, therapy approaches have evolved to accommodate molecular diversity, with a focus on more biologically based therapies to minimize negative consequences. To regulate cell fate in human breast cells, the Hippo signaling pathway has been associated with the alpha subtype of estrogen receptors. This pathway regulates tissue size, regeneration, and healing, as well as the survival of tissue-specific stem cells, proliferation, and apoptosis in a variety of organs, allowing for cell differentiation. Hippo signaling is mediated by the kinases MST1, MST2, LATS1, and LATS2, as well as the adaptor proteins SAV1 and MOB. These kinases phosphorylate the downstream effectors of the Hippo pathway, yes-associated protein (YAP), and transcriptional coactivator with PDZ-binding motif (TAZ), suppressing the expression of their downstream target genes. The Hippo signaling pathway kinase cascade plays a significant role in all cancers. Understanding the principles of this kinase cascade would prevent the occurrence of breast cancer. In recent years, small noncoding RNAs, or microRNAs, have been implicated in the development of several malignancies, including breast cancer. The interconnections between miRNAs and Hippo signaling pathway core proteins in the breast, on the other hand, remain poorly understood. In this review, we focused on highlighting the Hippo signaling system, its key parts, its importance in breast cancer, and its regulation by miRNAs and other related pathways.

## Introduction

Breast cancer is the most frequent female cancer and is a molecularly heterogeneous disease. It is frequently treated with chemotherapy and hormone therapy.^1^^,^^2^ Breast cancer develops as a result of aberrant cell or tissue proliferation within the breast glands.^3^ The majority of breast malignant tumors are carcinomas, mainly adenocarcinomas. These cancer intrinsic subtypes are biologically different entities with diverse natural gene expression patterns. Luminal A, Luminal B, HER2-enriched, and Basal-like 3 are the most generally recognized intrinsic subtypes. Breast cancer is a heterogeneous disease that comprises tumor initiation and growth, metastasis and invasion, and angiogenesis.^4^ Therapy approaches have changed in recent years to account for molecular variation, with an emphasis on more biologically based treatments to prevent unwanted effects.^5^

A number of signaling pathways, including Wnt/β-catenin, Hedgehog (Hh), Hippo, MAPK/ERK, and PI3K/PTEN/AKT, are involved in normal breast development.^6^, ^7^, ^8^, ^9^ Furthermore, new research shows that non-coding RNAs and epigenetic regulators possibly play a major role in the development of breast tumors and contribute to breast cancer gene diversity and metastatic features, particularly in triple-negative breast cancer.^10^ Alterations in these pathways are strongly connected with human cancer, particularly breast cancer.^11^ Recognizing the biology of breast cancer helps determine which anticancer agents are most effective.^1^

The Hippo signaling pathway was found in *Drosophila melanogaster*, a model organism to study cancer. This pathway has been connected to the alpha subtype of estrogen receptor to regulate breast cell fate in human breast cells.^12^ This pathway governs tissue size, regeneration and healing, and the survival of tissue-specific stem cells,^13^^,^^14^ proliferation, and apoptosis regulation in several organs, allowing for cell differentiation.^15^, ^16^, ^17^, ^18^ Hippo signaling is mediated by the kinases MST1, MST2, LATS1, and LATS2, as well as the adaptor proteins SAV1 and MOB.^19^^,^^20^ These kinases phosphorylate the downstream effectors of the Hippo pathway, YAP, and transcriptional coactivator with TAZ, and thus suppress the expression of their downstream target genes.^21^^,^^22^ Kinases in the Hippo signaling pathway cascade play critical roles in all malignancies. The Hippo pathway-dysregulated signaling has been connected to a variety of malignancies, namely breast, liver, lung, gastric, prostate, and colorectal cancers.^23^^,^^24^

Recently, non-coding RNAs have been widely recognized by evaluation of the function of the Hippo pathway in human cancers.^25^, ^26^, ^27^ Interestingly, worldwide transcriptome investigations revealed that just about two percent of the entire genome codes for proteins, with the vast majority non-coding.^28^ Non-coding RNAs (ncRNAs) constitute most of the human transcriptome and play a variety of essential regulatory roles in gene expression and other biological processes.^29^ Based on their size and structural or regulatory features, ncRNAs are divided into two types: long non-coding RNAs, or lncRNAs, and short non-coding RNAs, which include microRNAs (miRNAs), snoRNAs, and piRNAs.^30^ The miRNAs are the most widely investigated sncRNAs, with their biogenesis, activity, and role in cancer.^31^ Circular RNAs (circRNAs) and miRNAs are ncRNAs that have been linked to the development of cancer.^32^

miRNAs are regulators that serve as post-transcriptional repressors of gene expression. At the molecular level, they modulate CSC properties such as self-renewal, proliferation, differentiation, and fate.^33^ Additionally, miRNAs have appeared as important regulators of developmental processes. miRNA dysregulation leads to a variety of tumors, especially breast cancer.^29^

In this article, we focused on summarizing the Hippo signaling system, its key parts, its importance in breast cancer, and its regulation by miRNAs and other related pathways.

## The Hippo pathway is a developmental pathway

**Figure 1:**
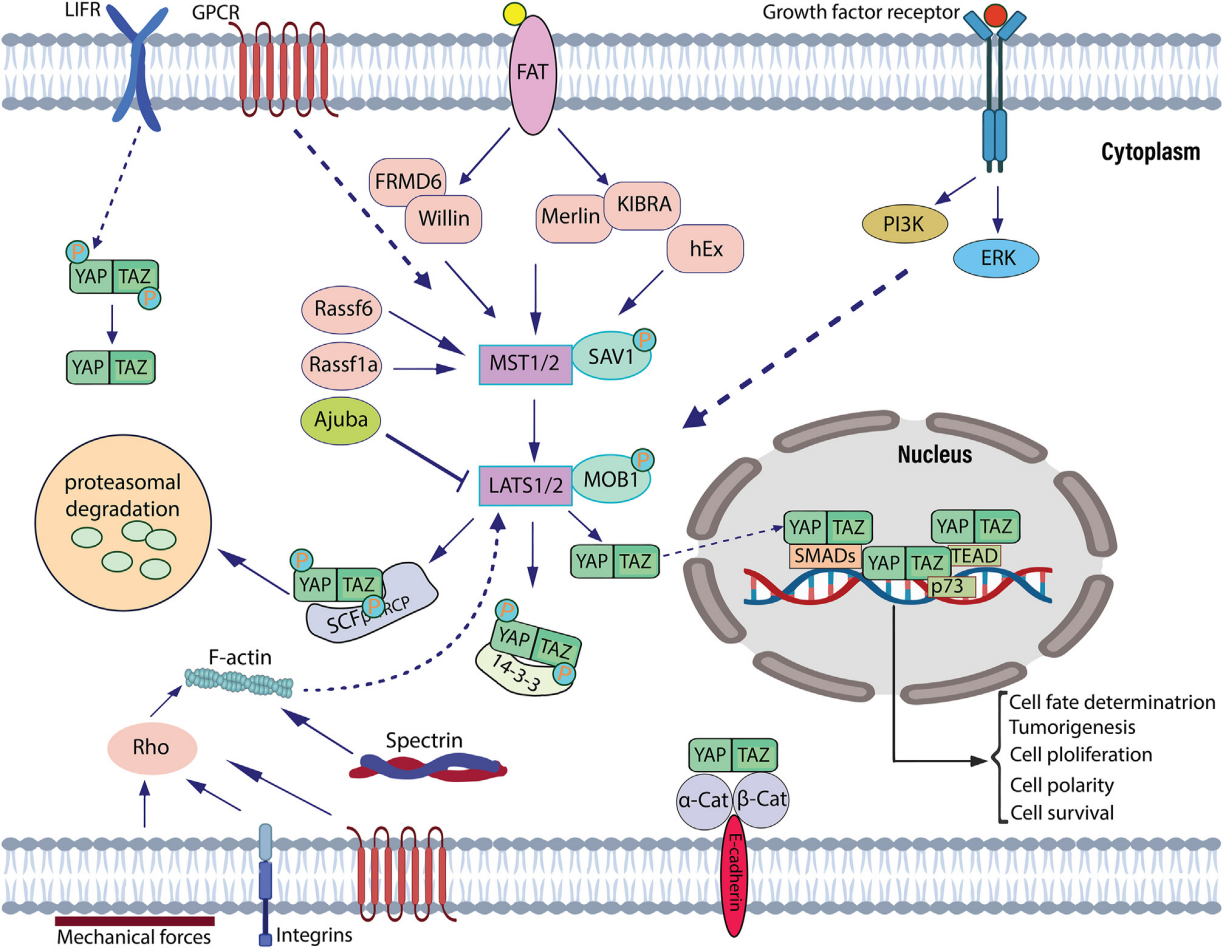
The Hippo signaling pathway and its interactions with other pathways. MST1, MST2, LATS1, and LATS2 phosphorylate YAP1 and TAZ, molecular components of the Hippo pathway. SAV1 and MOB1 are mediators that promote phosphorylation, leading to MST1 and MST2, as well as LATS1 and LATS2 closer together. TAZ and YAP phosphorylated molecules through interaction with 14-3-3 proteins and subsequently, TAZ and YAP are degraded by proteasomes due to the activities of the SCF complex. The unphosphorylated versions of YAP and TAZ stay in the nucleus and regulate certain transcription factors. The activity of MST1, MST2, LATS1, and LATS2 is altered as a result of interacting with proteins, notably NF2, KIBRA, RASSFs, and Ajuba proteins, leading to YAP1 and TAZ localization. G protein-coupled receptor ligands, MAPK, ERK, PI3K/AKT, JNK, Jak/Stat, and Wnt/β-catenin, have been demonstrated to interact with the Hippo pathway. Cytoskeletal rearrangement is triggered by physical forces that affect TAZ and YAP1.

LATS1 and LATS2 phosphorylate YAP1 and TAZ, molecular components of the Hippo pathway. SAV1 is a mediator that promotes phosphorylation, leading to MST1 and MST2, as well as LATS1 and LATS2 closer together. The kinase activity of LATS1/2 increased by two additional adapter molecules, MOB1. An activating cascade leads to the phosphorylation of 127 and 381 serine residues of YAP1 and 89 and 311 serine residues of TAZ as a result of LATS1/2-mediated phosphorylation. The TAZ and YAP1 phosphorylated molecules through interaction with 14-3-3 proteins and subsequent phosphorylation by CK1 kinase results in their degradation. TAZ and YAP are eventually degraded by proteasomes due to the activities of the Skp, Cullin, and F-box-containing complex (or SCF complex). The unphosphorylated versions of YAP1 and TAZ stay in the nucleus and are regulated by TEAD transcription factors.^34^^,^^35^ G protein-coupled receptor ligands were found to be one of several mechanisms that regulate the Hippo pathway,^36^ with GPER regulating estrogen activity in the development of breast cancer.^37^ The activity of MST1, MST2, LATS1, and LATS2 is altered as a result of interacting with proteins, notably NF2, KIBRA, and RASSFs, leading to YAP1 and TAZ localization.^38^^,^^39^ Cytoskeletal rearrangement is triggered by physical forces, and TAZ and YAP1 appear as central components between extracellular matrix signals and transcriptional outcomes that affect cellular responses.^40^^,^^41^ Additionally, other pathways, namely MAPK, PI3K/AKT, Jak/Stat, and Wnt/β-catenin, have been demonstrated to interact with the Hippo pathway.^42^^,^^43^ The stimulation of the Wnt/β-catenin pathway results in the separation of YAP1 and TAZ from the proteasomal degradation complex, which activates the Hippo pathway.^42^^,^^44^ The activation of the JNK pathway increased the dephosphorylation of YAP and the kinase involved in YAP phosphorylation.^43^^,^^45^ Moreover, Ajuba proteins inhibit LATS1/2 activity, which influences JNK Hippo pathway activation.^46^ Most importantly, in response to PI3K signaling stimulation, the Hippo pathway is inhibited, resulting in the modulation of tissue growth^47^ (Fig. 1).

### YAP/TAZ signaling downstream genes and targets

**Figure 2:**
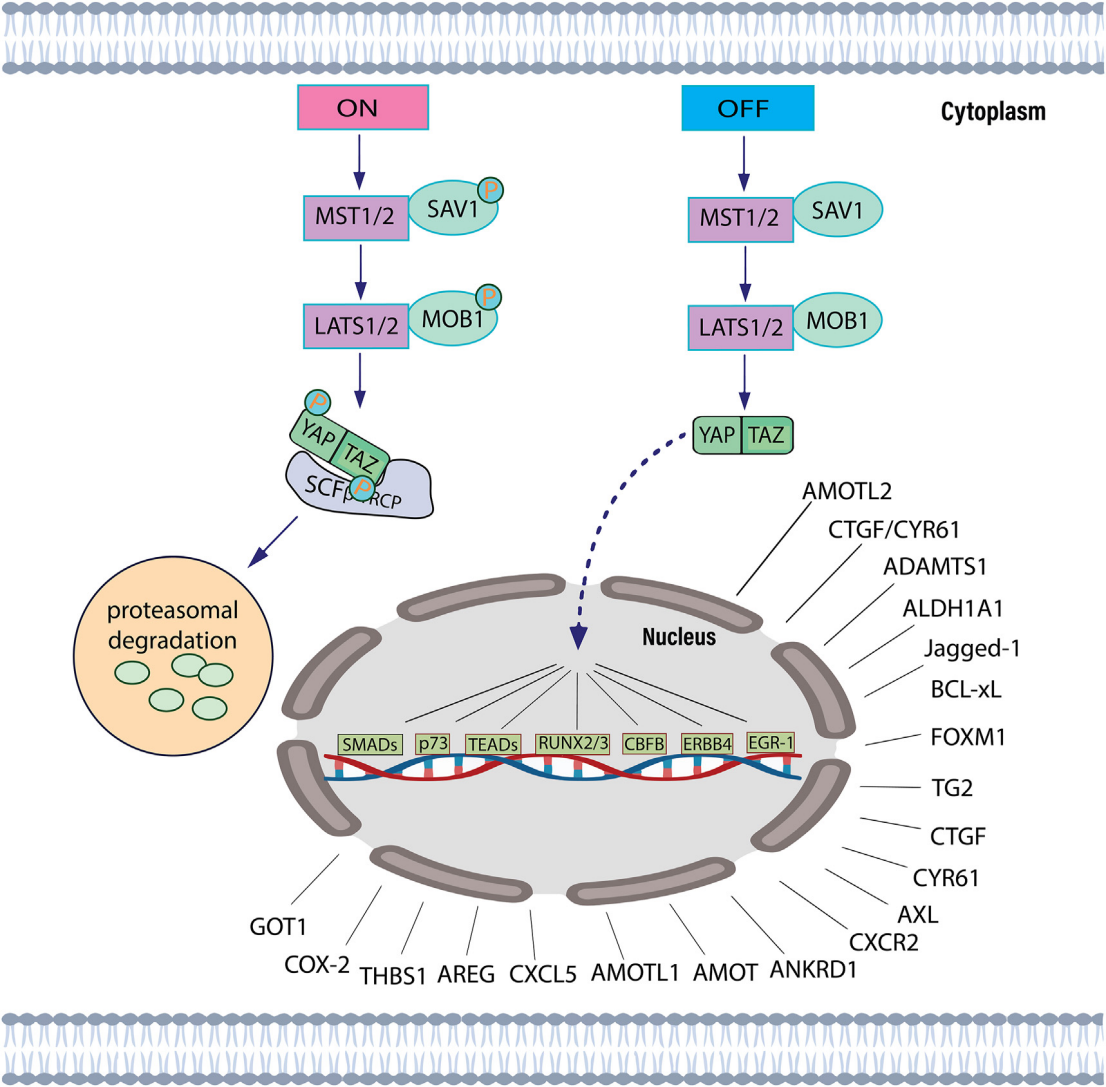
YAP/TAZ-downstream targets in the on and off states. The Hippo signaling pathway is active/ON (left). YAP/TAZ is not phosphorylated, relocates to the nucleus, binds to specific transcription factors such as TEADs, TP73, SMADs, RUNX2/3, CBFB, ERBB4, and EGR-1 regulates target genes necessary for cell proliferation, migration, and survival. The Hippo signaling pathway is inactive/OFF (right). Various signals lead to the phosphorylation of MST1/MST2, LATS1/LATS2, and YAP/TAZ. Phosphorylation of YAP/TAZ recruits 14-3-3 proteins that induce cytoplasmic retention or proteolytic cleavage.

Continuous cell proliferation, one of the most well-studied responses to the activation of YAP/TAZ, is actually gained by targeting genes involved directly in the cell cycle, such as *CCNE1*, *CDC25A*, and *CCNA2*, DNA replication and repair systems (*MCM7*, *MCM3*, *RAD18*, *CDC6*, *TOP2A*, *POLA2* and, *POLE3*), transcriptional factors (for example TEAD family), mitotic kinases, and associated factors.^48^^,^^49^ YAP/TAZ activation enhances chemoresistance and inhibits cancer cells from apoptosis by overexpression of *CTGF*, *CYR61*, *AXL* receptor tyrosine kinase genes, and *BCL-2* and the IAP family as anti-apoptotic genes.^50^^,^^51^ YAP/TAZ appears to utilize the TEAD family of transcription factors to trigger the majority of relevant gene expression processes.^52^ Certain YAP/TAZ-responsive genes are also influenced cooperatively by AP-1 transcription factors.^34^^,^^43^ In regard to interacting with TEADs, YAP/TAZ communicates with the mediator complex and chromatin modeling enzymes such as the methyltransferase and SWI/SNF complex to impact gene expression.^53^^,^^54^ Additional mechanism YAP/TAZ maintains enormous cell proliferation through modifying cell metabolism. YAP stimulates the metabolism of glucose by increasing the transcription of isoform 3 of the glucose transporter (GLUT3).^55^ YAP provides a continual source of glutamine for purine and pyrimidine by stimulating the production of glutamine synthetase.^56^ Additionally, YAP can activate the expression of the pathway of mTOR regulators, including RHEB, the mTORC1 activator, and LAT1^57^ (Fig. 2).

As a result, targeting YAP/TAZ-downstream specific targets is an interesting and promising potential cancer therapeutic approach. It is wonderful to know that several medications presently on the market effectively inhibit YAP/TAZ activity and that various YAP/TAZ inhibitors are under research.^58^

### microRNAs regulate Hippo pathway genes

miRNAs are short molecules with an average of 22 nucleotides in length that do not have the function of encoding a protein. miRNAs are involved in several physiological functions by attaching to the 3′UTR region of their targeted mRNAs to degrade target mRNAs or inhibit translational processes. In addition, miRNAs regulate a variety of cellular activities, namely proliferation, apoptosis, differentiation, and metastasis.^59^^,^^60^ There is significant evidence that miRNAs alter cellular mechanisms by modifying their target networks.^61^^,^^62^

miRNAs as new biomarkers in cancer treatment and early diagnosis have received attention in the last decade.^63^^,^^64^ OncomiR refers to a group of miRNAs that act as oncogenes and create an oncogenic phenotype. On the other hand, tumor suppressor miRNAs behave as anti-oncogenic molecules and limit the progression of cancer (Table 1). Furthermore, several miRNAs associated with components of the Hippo signaling pathway have been identified according to *in silico* research.^65^, ^66^, ^67^

**Table 1:** microRNAs that affect the Hippo pathway in breast cancer, either directly or indirectly.

miRNA symbol	Role in breast cancer	Targets	Mode of action	Reference
miR-135b	Oncogene	LATS2	miR-135b directly suppresses the LATS2 axis; miR-135b and the LATS2 axis suppress p-YAP and CDK2 expression	^3^,^79^,^80^
miR-93	Oncogene	LATS2	miR-93 suppresses LATS2 expression and thereby enhances tumor angiogenesis and metastasis	^81^
miR-372	Oncogene	LATS2	miR-372, via targeting LATS2, acts as an oncogenic microRNA in breast cancer	^82^
miR-520b	Anti-oncogene	LAST2	Local expression of miR-520b increases the stemness of tumor cancer cells by down-regulation of LAST2	^83^
miR-424	Anti-oncogene	LATS1	Expression of miR-424 stimulates proliferation and invasion of cancer cells by targeting the LATS1 gene, and inhibits cell growth and arrest in cell cycle phases in human breast cancer by negatively regulating CDK1 mRNA	^84^,^119^
miR-125a-5p	Oncogene	LIFR and TAZ	Circ_0001667 and hsa_circ_0052112 act as sponges for miR-125a-5p, leading to the inhibition of TAZ action by targeting LIFR and promoting cell migration and invasion in breast cancer	^87^,^120^
miR-515-5p	Oncogene	YAP and TAZ	LINC00673 and LINC00519 act by sponging miR-515-5p to up-regulate YAP1	^91^,^121^
miR-200a-3p	Oncogene	YAP	hsa_circ_0005273 up-regulates YAP by sponging miR-200a-3p	^92^,^122^,^123^
miR-1297	Anti-oncogene	TAZ	Overexpression of miR-1297 decreases TAZ expression	^32^,^124^
miR-30a	Anti-oncogene	YAP	miR-30a drastically decreases the expression of YAP in cancerous cells	^67^
miR-182	Oncogene	SMAD7	miR-182 inhibits SMAD7 transcription and deactivates the TGF-β negative feedback loop	^93^
miR-106b-25	Oncogene	SMAD7	miR-106b-25 regulates SMAD7, resulting in an EMT dependent on Six1 and cancer-starting cell morphology	^95^,^97^
miR-375	Anti-oncogene	YAP	miR-375 promotes treatment responses in 5FU-resistant cells via stimulating the Hippo pathway through YAP1	^99^
miR-181	Anti-oncogene	YAP and TAZ	miR-181c stimulates YAP/TAZ, which leads to the activation of subsequent genes	^100^,^105^
miR-326	Anti-oncogene	TAZ	Circ_0000511 up-regulates TAZ levels by sponging miR-326 in breast cancer cells	^106^
miR-574-5p	Oncogene	TAZ	BCL11A and SOX2 are miR-574-5p targets that suppress TAZ	^107^
miR-205	Oncogene or Anti-oncogene	TAZ	miR-205 reduced TAZ in breast epithelial stem cell aggregates as well as acting as an anti-oncogene by inhibiting ErbB3, VEGFA, and ZEB1/2 in breast, melanoma, and lung cancers.	^113^
miR-506	Anti-oncogene	YAP	miR-506 suppresses cell growth and disrupts the cell cycle by targeting YAP	^125^,^126^
miR-223-3p	Oncogene	Yap and LATS1	miR223-3p suppresses Yap1 phosphorylation and LATS1 protein expression	^115^
miR-9	Oncogene	CDH1 and LIFR	miR-9 enhances metastasis by targeting the tumor suppressor genes CDH1 and LIFR	^13^,^117^

miRNA content, trafficking, and quantity were altered in cancer cells. In this way, circulating miRNAs may serve as cancer biomarkers.^68^ Many mRNAs can be regulated by a single miRNA, resulting in transcriptional regulation.^69^ miRNAs can act as oncomiRs or tumor suppressors depending on their target genes and the conditions.^70^ miRNAs have recently been discovered to act as ligands to activate several signaling pathways.^71^^,^^72^

Using specific selection criteria, researchers discovered 69 highly related, repeatable mRNA-miRNA regulatory associations between five Hippo core genes and 18 miRNAs across nine types of cancer.^17^ The microRNA-200 subfamily is well known as mRNA regulators. They are well recognized for their roles in epithelial-to-mesenchymal transition (EMT) regulation, and they have been shown to suppress YAP1 in human breast tumor cells.^1^^,^^16^ In addition, several miRNAs have been described with significant experimental evidence for being effectors of TAZ in the Hippo signaling pathway. They discovered that miR-590-3p and miR-200a-3p considerably reduced the expression of TAZ at the mRNA and protein levels.^1^ In a systematic review article, Wang et al report several regulatory miRNAs for Hippo pathway genes and indicate that relevant miRNA analogs could be used clinically.^17^

Previous research has shown that circRNAs have the potential to behave as miRNA sponges, regulating miRNA and its downstream targets. The findings revealed a circular RNA signature in hypopharyngeal cancer, implying that a central part of the ceRNA-miRNA network, which regulates the ErbB and Hippo pathways, plays a key role in Hca progression.^73^

### Interactions between microRNAs and the Hippo signaling pathway

**Figure 3:**
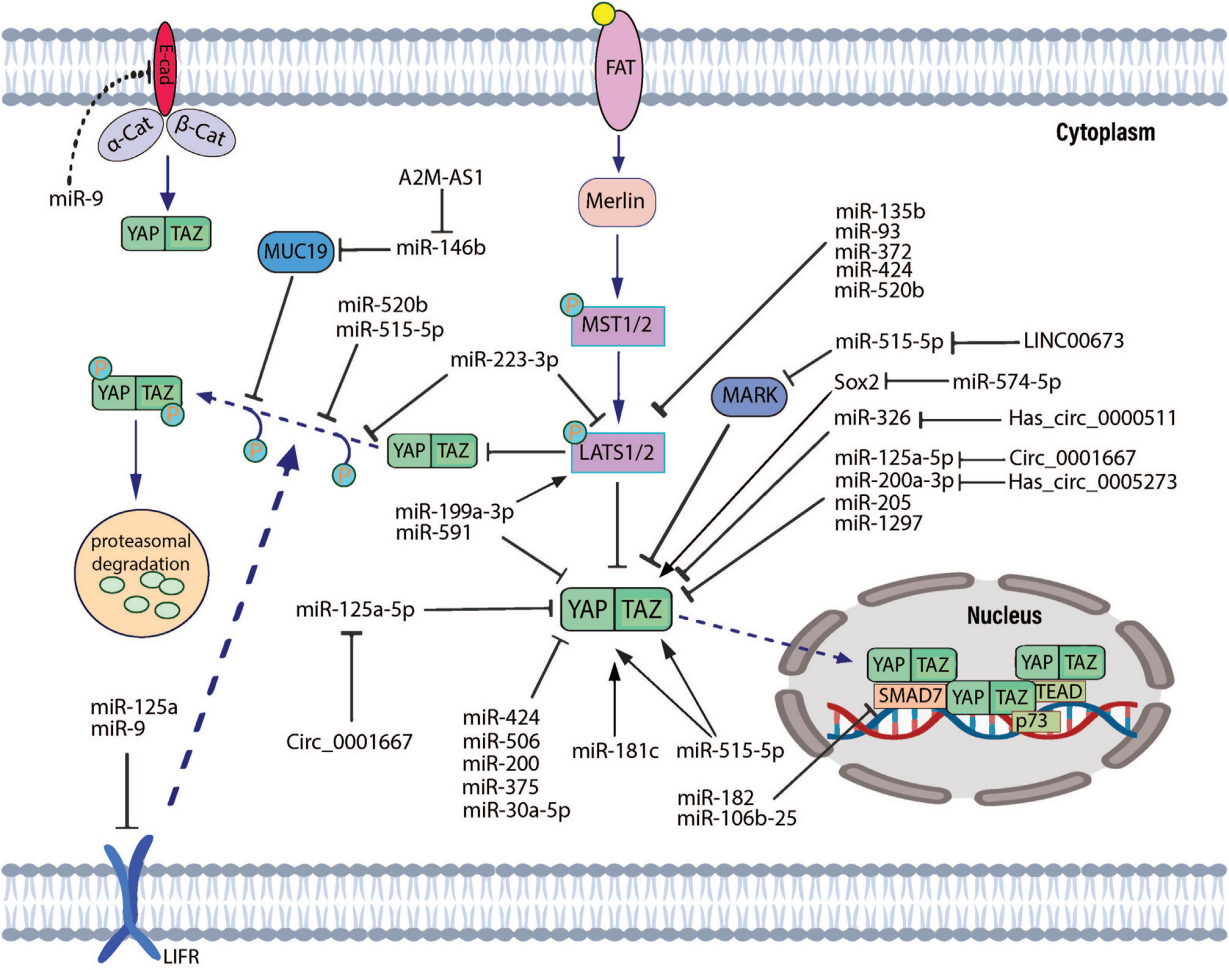
Interaction between microRNAs and the Hippo signaling pathway in breast cancer. These interactions exist in different parts of the pathway, including regulation of core proteins, LATS1/LATS2, YAP/TAZ, and phosphorylation of YAP/TAZ. Additionally, miR-9 and miR-125a have an influence on downstream effectors as well as LIFR and E-cadherin, which indirectly impact the signaling pathway.

miRNAs may have different functions during cancer progression via direct or indirect influence on YAP, TAZ, MST1, MST2, LATS1, and LATS2 in the Hippo pathway. For instance, miR-125a-5p shows a decrease in proliferation and stimulates differentiation in retinoblastoma by affecting TAZ.^74^^,^^75^ In contrast, miR-125a-5p inversely induced the expression of TAZ in breast cancer cells by suppressing leukemia inhibitory factor receptor (LIFR), an inhibitor of YAP and TAZ activity that raises the fraction of stem cells in cancer.^76^ Additionally, miR-9 stimulates breast tumor cell migration and metastasis by straightforwardly affecting LIFR, which reduces YAP1 activity,^77^ whereas miR-9-3p serves as a cancer repressor in hepatocellular carcinoma via targeting TAZ, which stimulates cell proliferation^78^ (Fig. 3).

### The microRNAs regulate the activity of LATS1 and LATS2

Hua et al show that miR-135b expression is typically elevated in breast cancer tissues and cells. Cellular proliferation, migration, and invasion, as well as the cell cycle of breast cancer cells, are influenced by miR-135b-induced overexpression. Further research indicates how miR-135b and the LATS2 axis can influence p-YAP and *CDK2* as downstream genes.^3^^,^^79^^,^^80^ Overall, their data suggest that the miR-135b/LATS2 axis could be a future therapeutic target for breast cancer.

Increased levels of LATS2 in the tumor tissue were connected to apoptosis. The miR-93 is a target of LATS2, according to the study by Fang et al. LATS2 silencing increased cell survival, invasion, and tube formation, whereas LATS2 overexpression inhibited cell survival and invasion. The authors found that miR-93 suppressed LATS2 expression and thereby enhanced tumor angiogenesis and metastasis. Their findings indicate that inhibiting miR-93 function might be a viable strategy for suppressing tumor spread.^81^

Down-regulation of miR-372 significantly reduced cell proliferation, blocked the cell cycle in the G1/S phase, and boosted apoptosis in breast cancer cells. An *in vivo* experiment confirmed the tumor-inhibitory impacts of miR-372 knockdown. Later research found that miR-372 affected the expression of LATS2 in breast cancer cells by directly targeting its 3′-untranslated region. Additionally, suppressing LATS2 might reverse the impact of the miR-372 inhibitor. Therefore, the findings imply that miR-372, via targeting LATS2, acts as an oncogenic miRNA in breast cancer.^82^

The miR-520b has been found to be overexpressed in breast cancer stem cells. Local expression of miR-520b improves the stemness of breast cancer cells, whereas inhibition of miR-520b reduces stemness. The Hippo signaling pathway is effectively regulated by miR-520b, and up-regulation of LAST2 reduced the impact of miR520b on the stemness of tumor cancer cells.^83^

The expression of miR-424 was lower in human mammary tumors as well as cell lines when compared to non-malignant tissues and cells. According to a study, miR-424 may inhibit cell growth and arrest in cell cycle phases (particularly G2/M) in human breast cancer by negatively regulating cyclin-dependent kinase 1 (CDK1) mRNA, which may occur via the Hippo and ERK pathways.^84^ Ras, Raf, MEK1/2, and ERK1/2 are important components of the ERK pathway, which regulates cell proliferation, distant metastasis, and differentiation.^85^^,^^86^ They actively participated in the transformation of cancerous cells. Findings predicted that the miR424/CDK1 axis changed ERK1/2 activation.^84^

### microRNAs that regulate the activity of YAP and TAZ

Circ_0001667 inversely affects TAZ through sponging miR-125a-5p, which enhances cancer cell proliferation and metastasis.^87^ The function of LIFR was discovered to be tightly tied to miR-125a repression and up-regulation.^76^ miR-125a inhibits the action of the Hippo signaling mediator molecule TAZ by targeting LIFR.^88^ Additionally, in breast cancer cells, miR-125a regulates stem cells through Hippo signaling suppression by LIFR.^76^ According to current research, long intergenic non-protein coding RNA 673 (LINC00673) is a candidate repressor for which variant is related to a greater risk of pancreatic cancer.^89^ On the other hand, LINC00673 has been implicated in tumorigenesis in a number of malignancies.^90^ LINC00673 acts by sponging miR-515-5p to regulate MARK4 and then blocking the Hippo signaling pathway to increase tumor development.^91^

Researchers discovered that has_circ_0005273 is a circRNA generated from several exons of PTK2 and that it was strongly overexpressed in breast cancer tissues and cell lines. Has_circ_0005273 played an oncogenic effect in breast tumors by behaving like a sponge for tumor repressor miR-200a-3p, which inactivated the YAP1-Hippo signaling pathway. Has_circ_0005273 accelerates breast cancer progression via regulating the miR-200a-3p-YAP1-Hippo signaling axis and inactivating the axis, which might be employed as a biomarker and therapeutic target.^92^

The expression levels of has_circ_00Qiao074 and miR-1297 in breast cell lines have an inverse association, which is the strongest in cancer cell lines and the lowest in normal lines. Silencing has_circ_0091074 increased the quantity of miR-1297, suggesting a negative relationship between has_circ_0091074 and miR-1297. According to Hu et al, miR-1297 suppresses breast cancer cell proliferation and invasiveness of breast cancer cells, suggesting the hsa_circ_0091074/miR-1297/TAZ/TEAD4 axis could be a therapeutical approach.^32^

miR-30a targets the oncogene YAP, which is frequently dysregulated in breast cancer and is associated with a poor prognosis.^65^, ^66^, ^67^ Researchers discovered a significant inverse association between miR-30a and YAP in MCF-10A cells (as normal) and MDA-MB-231 (as cancerous) cells using Real-Time Quantitative-PCR. They indicate that miR-30a drastically decreases the expression of YAP in cancerous cells compared to normal cells. The findings might assist in understanding whether miR-30a expression leads to a better prognosis in triple-negative breast cancer patients.^67^

TGF-β signaling increases the expression of miR-182, which inhibits SMAD7 transcription and deactivates the TGF-β negative feedback loop during malignancy.^93^ The miR-182 up-regulation increases breast cancer invasion and TGF-β-induced osteoclastogenesis, facilitating bone metastases. Moreover, in patient tumor tissue samples, the expression of miR-182 is negatively related to that of the SMAD7 (SMAD Family Member 7) molecule. As a result, findings show that miR-182-mediated TGF-β self-restraint is disrupted and propose a strategy to explain the TGF-β responses in metastatic cancer cells.^94^

In breast cancer cells, the miR-106b-25 regulates SMAD7, resulting in an EMT dependent on Six1 and cancer-starting cell morphology. The miR-93 and miR-106b concentrations in breast tumors are significantly associated with Six1 and TGF-β signaling.^95^ Scientists suggested that inhibiting miR-93 concentration might suppress breast cancer metastases.^96^ Nuclear localization of SMADs (TGF- transcriptional effectors) is required for TGF signaling. YAP and TAZ are transcription factors of the Hippo pathway and regulate the activity of SMADs. These findings showed that miR-106b influenced breast cancer development.^97^

miR-375 was shown to suppress the expression of YAP and its target CCN2 (or connective tissue growth factor). Furthermore, miR-375 overexpression in HCC cells decreased cell growth and invasion.^98^ For the first time, Xu et al showed that miR-375 dramatically promotes treatment responses in cells resistant to 5FU via stimulating the Hippo pathway through YAP1. A variety of anticancer medicines, such as capecitabine, 5FU, irinotecan, and oxaliplatin, were examined for similar suppression of drug resistance by miR-375. The HCT116/FU xenograft assay revealed a similar effect on chemoresistance *in vivo*, confirming miR-375 as a viable target for multiple forms of resistance to drugs.^99^

The family of miR-181 is organized into miR-181a, b, c, and d, each of which is supplementary to a wide variety of mRNAs.^100^ This family of miRNAs has been recognized as the most significant miRNA family in cancer prognosis, with the potential to serve as novel therapeutic strategies for beneficial tumor treatments. Inappropriate miR-181 family transcription has also been associated with a multitude of malignancies, such as gastric cancer, breast cancer, and lung cancer.^101^, ^102^, ^103^ miR-181c has been shown to be highly expressed in inflammatory breast cancer and to suppress cancer growth by targeting the gene-PTEN mRNA.^104^ According to the research, increased levels of miR-181c in the Hippo pathway stimulate YAP/TAZ, which leads to the activation of subsequent genes, including *CTGF*, *BIRC5*, and *BLC2L1*, contributing to higher pancreatic cancer cell proliferation.^105^ Down-regulation of miR-181c in malignant MCF-7 cells leads to increased doxorubicin chemoresistance compared to normal cells. *In vitro* and *in vivo*, increased up-regulation of miR-181c strongly suppressed cell proliferation and hypersensitivity to chemotherapy by doxorubicin and the generation of resistant breast cancer xenograft tumors.^100^

circ_0000511 concentrations were found to be unusually high in breast cancer samples and cell lines as compared to normal controls. circ_0000511 inhibited breast cancer cell proliferation, migration, and invasion while inducing apoptosis through up-regulating TAZ levels by sponging miR-326 in breast cancer cells. circ_0000511 increased TAZ expression in breast cancer cells by targeting miR-326. The circ_0000511/miR-326/TAZ axis might be a potential target for the therapy of breast cancer.^106^

The impact of miR-574-5p on TNBC carcinogenesis revealed that miR-574-5p amounts in breast cancer tissues and cells were reduced. The miR-574-5p suppressed proliferation, migration, and EMT in TNBC cells. In addition, miR-574-5p also suppressed tumor development and metastasis *in vivo*. Furthermore, miR-574-5p inhibited tumor growth and metastasis *in vivo*. BCL11A and SOX2 are targets of miR-574-5p that suppress the SKIL/TAZ/CTGF axis. The regulatory impact of miR-574-5p in TNBC cells was partially related to SOX2 and BCL11A. Furthermore, SOX2 regulation of downstream oncogenes is dependent on BCL11A. It suggests that TNBC carcinogenesis is mediated by the miR-574-5p/BCL11A/SOX2 axis, offering a new approach to understanding TNBC progression.^107^

Scientific evidence supports the dual functions of miR-205 as a tumor suppressor by affecting ErbB3, VEGFA, and ZEB1/2 in breast, melanoma, and lung cancers, as well as an oncogene by influencing PTEN, TRAF2, and SHIP2 in breast cancer, nasopharyngeal carcinoma, and lung squamous cell carcinoma.^108^ miR-205 disrupts ZEB1/2-mediated EMT^109^ and inhibits tumor development from the basal membrane to the stroma.^110^

Mayoral-Varo et al utilized the SUM159PT cell line to investigate the impact of ectopic miR-205 expression on breast cancer development. SUM159PT cells originated from a human primary anaplastic breast cancer.^111^^,^^112^ These cells are thought to be an excellent model of TNBC cells. They observed that miR-205 suppressed the expression of CD44 and TAZ, E2A.E12, Twist, Snail1, and CK5, all of which are involved in EMT. Interestingly, although multiple mesenchymal markers were decreased in SUM159 miR-205, E-cadherin expression was not observed. They discovered that miR-205 suppressed cell proliferation, migration, invasion, anchorage-independent growth, and, most significantly, the self-renewal of tumor-initiating/cancer-stem cells. These findings imply that miR-205 could potentially influence SUM159 invasion and metastasis by suppressing cancer stem cell renewal.^113^

According to the above studies, miR-205 functions as a tumor suppressor by reducing cancer cell proliferation or as an oncogene by stimulating cancer development, depending on which target gene it affects.

### Microarrays affecting YAP/TAZ phosphorylation

According to research, the function of lncRNAs as competitive endogenous RNA (CeRNA) affects breast cancer progression by regulating miRNAs. The expression levels of miR-146b were much lower than lncRNA A2M-AS1 and MUC19 levels in breast cancer cells as well as tissues. A2M-AS1 down-regulation decreased breast cell proliferation, colony formation, and invasion, and improved apoptosis. Furthermore, negative expression of miR-146b regulated by A2M-AS1 in breast cancer cells was also observed. Dual-luciferase reporter technique confirmed that the miR-146b targets the 3-UTR of *MUC19*. Additionally, MUC19 regulates the function of the Hippo signaling pathway via miR-146b and A2M-AS1.^114^

The expression levels of miR-223-3p in breast cancer cells were shown to be considerably greater than in normal cells. Furthermore, when a miR-223-3p inhibitor was introduced into cells, tumor cell activity was significantly decreased, and apoptosis was increased. Hence, miR-223-3p has a major impact on the proliferation, invasion, and metastasis of breast cancer cells. The results of Western blotting indicated that inhibiting miR223-3p raised Yap1 phosphorylation and LATS1 protein expression levels.^115^ Moreover, miR-520b and miR-515-5p greatly reduced the phosphorylation of YAP in breast cancer cells.^83^^,^^91^

### Other microRNAs that influence the Hippo signaling pathway

E-cadherin dysregulation or loss of function has been associated with human cancers.^116^ LIFR is down-regulated in human breast cancer and is inversely connected with metastasis. miR-9 enhances metastasis by targeting the tumor suppressor gene E-cadherin and LIFR,^13^^,^^117^ especially in breast cancer cells.^77^ LIFR is down-regulated in human breast cancer and is inversely connected with metastasis. Increasing LIFR in cancerous cells inhibits metastasis via activating a Hippo signaling kinase that causes the transcriptional coactivator YAP to be phosphorylated, remain in the cytoplasm, and functionally inactivated. On the other hand, lack of LIFR induces migration, invasion, and metastases in nonmetastatic breast cancer cells via activating YAP.^77^

## Conclusions and future perspectives

Breast cancer is the most common female malignancy and a molecularly heterogeneous disease. In recent years, therapeutic approaches have shifted to account for molecular variation, with an emphasis on more biologically based treatments to avoid side effects, and breast cancer is no exception. Understanding the mechanisms of novel processes and their association with biological molecules should be properly studied in order to achieve improved and more effective strategies. As a result, it is critical to evaluate the Hippo signaling pathway as a signaling pathway that has recently been investigated, as well as the unquestionable significance of miRNAs in regulating cancer-related signaling pathways, particularly in the Hippo signaling pathway. The Hippo signaling pathway kinase cascade plays critical roles in all malignancies. The Hippo pathway-dysregulated signaling has been connected to a variety of malignancies, namely breast, liver, lung, gastric, prostate, and colorectal cancers. Additionally, miRNAs have appeared as important regulators of developmental processes. miRNA dysregulation leads to a variety of tumors, especially breast cancer. There is no doubt that miRNAs play an important role in the initiation and development of several malignancies, mainly breast cancer. Due to their simplicity of identification and strong stability, this class of non-coding RNAs is highly suited for application in biomedicine and cancer biology. Some of these molecules may differ in their activity between cancers. Some miRNAs, on the other hand, appear to be more prevalent in certain cancers, whereas others appear to be slightly prevalent. For instance, miR-125a-5p shows a decrease in proliferation and stimulates differentiation in retinoblastoma by affecting TAZ.^74^^,^^75^ In contrast, miR-125a-5p inversely induced the expression of TAZ in breast cancer cells by suppressing LIFR, an inhibitor of YAP and TAZ activity that raises the fraction of stem cells in cancer. Furthermore, miRNAs, such as miR-205, have a dual function as a tumor suppressor by affecting ErbB3, VEGFA, and ZEB1/2, as well as an oncogene by influencing PTEN, TRAF2, and SHIP2 in breast cancer, which could lead to confusion among researchers in this field. In this current situation, it is critical to evaluate and review the leading role of each miRNA at different stages of cancer and across various pathways. Further research in these areas should also be conducted.

The findings demonstrate that these miRNAs might be employed as prognostic, diagnostic, and therapeutic biomarkers for breast cancer. miRNA-based therapies have previously been employed as critical strategies in the treatment of breast cancer.^118^ Therefore, further research into the impact of miRNAs on breast cancer is required. In this article, we summarized the Hippo signaling pathway, its key parts, its regulation by miRNAs and other related pathways, and its importance in breast cancer.

## Conflict of interests

The authors declare no potential conflict of interests.
